# Trends in Finnish Public Orthodontic Care from the Professionals' Perspective

**DOI:** 10.1155/2009/945074

**Published:** 2009-02-01

**Authors:** Ilpo Pietilä, Terttu Pietilä, Juha Varrela, Pertti Pirttiniemi, Pentti Alanen

**Affiliations:** ^1^Oral Health Services, Health Centre of Pori, P.O. Box 33, 28601 Pori, Finland; ^2^Departments of Oral Development and Orthodontics and Public Health Dentistry, Institute of Dentistry, University of Turku, 20520 Turku, Finland; ^3^Department of Oral Development and Orthodontics, Institute of Dentistry, University of Oulu, 90014 Oulu, Finland

## Abstract

The study maps out orthodontic care in Finnish municipal health centres in 2001, describes changes during the previous ten years reported by chief dental officers, and assesses the views of orthodontists on current public orthodontic services. The data were collected by questionnaires sent to all health centres and all orthodontists in Finland. Of all 0–18-year-olds, 11% were receiving orthodontic treatment with an appliance (range 2–43% among the health centres). The most frequently used appliances were headgear, quadhelix, and fixed appliances. Limited economic resources and the lack of orthodontic expertise were mentioned by the chief dental officers as factors decreasing the volume of services. The orthodontists mentioned the large regional variation and the lack of national guidelines as the most important aspects that should be improved on a national basis. To bring about improvement, they suggested increasing the number of specialist orthodontists and the delegation of orthodontic tasks to auxiliaries.

## 1. Introduction

In Finnish health centres, orthodontic treatment is an important part of children's and adolescents' dental services, all of which are free of charge up to the age of 18 years. In the early 1990s, every fourth dental visit of children and adolescents to the health centres was connected with orthodontic treatment [1]. However, during the economic depression later in the1990s, most health centres had to restrict their expenditure. At the same time, the focus in public dental health care was gradually changing, and today, dental care for adults of all ages is also included in the services. Despite this development, it is generally accepted that children's dental services should not be endangered. Each municipal health centre can decide on the extent of services they want to deliver. Consequently, the access to orthodontic treatment varies considerably [2, 3].

In countries with publicly funded orthodontic services, dentists play a dominant role in the initiation of orthodontic treatment [4, 5]. The access to orthodontic treatment is influenced by two main factors; the rates of referrals to orthodontists for assessment and the sufficiency of services. According to Shaw et al. [6], the referral rate is influenced by the thoroughness of examination, the consistency of the assessment of treatment need, and the perceived efficacy of treatment. Different guidelines have been developed for the assessment of orthodontic treatment need, but they do not seem to have much impact on the practices of the general dental practitioners [7]. In Finland, the most frequently applied method in the assessment of treatment need is a 10-grade scale modified from Grainger's TPI-index [8] by Heikinheimo [9] that is used in the majority of health centres. On the scale, grade ten represents the most severe malocclusions or craniofacial malformations, and grade one no malocclusion. The most frequently applied cut-off level entitling to treatment is 7. However, in different health centres the cut-off level varies between grades 2 and 8 according to the resources of the health centre [1].

The availability of services is greatly influenced by the distribution of orthodontic manpower. In most western countries, the majority of specialists live in the largest cities or in the most densely populated areas. In sparsely populated areas, this may lead either to restrictions in access to orthodontic treatment or to orthodontic treatment given by general dentists [10]. In Finland, the availability of specialist expertise varies in different areas of the country. In most health centres, the role of specialist orthodontists is central in diagnostics and treatment planning, but general dentists commonly provide treatment at least in all centres other than the largest health centres [2].

When public orthodontic care of children and adolescents is evaluated, it is important to study both the changes in orthodontic services as such and the perspective of the entire dental health care. The aim of this study was to examine orthodontic care in Finnish municipal health centres in 2001, and to assess the prevailing state and development during the past ten years as viewed by the local chief dental officers and the orthodontic specialists working in the health centres.

## 2. Methods

In April 2002, two different semistructured questionnaires were sent out to survey the views on orthodontic care in Finland. A questionnaire was sent to all local chief dental officers in 276 Finnish municipal health centres. The questionnaire was based on an earlier questionnaire, which mapped out the orthodontic care in Finnish health centres in 1992 [2], and it inquired about the number of personnel involved in orthodontic care, their work division, the number of orthodontic patients and visits, the use of removable and fixed orthodontic appliances, changes in orthodontic care in the previous ten years, and the chief dental officers' own view on what further orthodontic research is needed. A follow-up letter was sent to the chief dental officers who did not respond by the appointed time.

Another different questionnaire was sent to all 146 specialist orthodontists under 65 years of age living in Finland in 2001, regardless of their type of employment. The names and addresses of the orthodontists were obtained from the files of the Finnish Dental Society. The answers received concerning the optimal timing of orthodontic treatment, the main indications for starting treatment in children at each developmental stage of occlusion, and the respondents' choice of appliances have been analyzed in a previous study [11].

The present study includes only the answers of those respondents who worked in the health centres as salaried specialists or consultant orthodontists, or who provided commissioned orthodontic services for health centres, because they possessed real facts about activities in health centres instead of just beliefs. The orthodontists were asked to evaluate, in open questions, orthodontic services and optimal work division in municipal health centres, to report recent changes in their treatment practices, to give suggestions for improvement of orthodontic care, and to suggest orthodontic issues needing further research.

Responses were received from 177 chief dental officers, and after a follow-up letter, from a further 30 respondents. The total response rate was 76%. The nonresponding chief dental officers worked mainly in small health centres covering areas with fewer than 10 000 inhabitants. Six nonresponding chief dental officers worked in health centres covering areas with 10 000–20 000, another six with 20 000–50 000, and one with over 50 000 inhabitants.

In all, a response was received from 83 specialist orthodontists. Thirteen of them had no connection with health centres and were excluded from the study; 70 respondents working in or cooperating with health centres were included in the study. Twenty-two of the 63 nonrespondents worked in the health centres as salaried orthodontists, and ten cooperated as consultant orthodontists with the health centres. The response rate was 68%, when only the respondents and nonrespondents working in or cooperating with the health centres were included.

## 3. Results

### 3.1. Orthodontic care in municipal health centres in 2001

Orthodontic services were provided in all the responding health centres. The volume of orthodontic services was measured by the percentage of 0–18-year-olds wearing orthodontic appliances in 2001, the mean percentage being 11.4 (SD 6.4, range 2–43%). The mean percentage of children wearing an appliance was the highest in small health centres with fewer than 10 000 inhabitants. The mean percentage was slightly smaller in larger centres, but the differences were not statistically significant. The percentage of orthodontic visits of all dental visits of the 0–18-year-olds was, on average, 30.7 (SD 10.6, range 2–66% among health centres), and the size of the health centre was not associated with the ratio of orthodontic visits (Table 1).

A quadhelix was the most frequently used appliance in primary dentition, followed by an eruption guidance appliance. A headgear was the most frequently used appliance both in the age group of 7 to 9 years, and in the age group of 10 to 13 years (Table 2).

The most frequent way to obtain orthodontic expertise, used by 74% of health centres, was by making a contract with a consultant orthodontist. Every fifth health centre had employed salaried orthodontist manpower. Commissioned services were purchased in 34% of health centres. The purchasing of commissioned services was most frequent in the small health centres with fewer than 20 000 inhabitants. Five percent of health centres did not have any specialist expertise at their disposal. The ways of obtaining orthodontic expertise in the health centres of different sizes are given in Table 3. Specialist orthodontists' working time represented 22% of the total working time spent on orthodontic treatments in all the responding health centres.

In almost all the health centres (94%), general dentists treated some of the orthodontic patients. The working time they spent on orthodontic treatments represented 64% of the total working time spent on orthodontic care in the health centres. Delegation of orthodontic tasks to dental auxiliaries was used in 61% of health centres, and their working time represented 14% of the total working time spent on orthodontic care in the health centres.

Seventy-four percent of chief dental officers reported major changes in the organization of orthodontic services during the previous five years. In thirty-four health centres, major changes had taken place in the volume of orthodontic services (Table 4). Most of these changes concerned orthodontic specialist services. The number of specialist orthodontists had increased in twenty-seven and decreased in seven health centres.

### 3.2. Specialist orthodontists' views on orthodontic care in health centres

The specialist orthodontists proposed that specialists should not give simple orthodontic treatment but concentrate on treatment planning, consultation, and difficult treatment. Forty-five respondents (64%) wanted to change the work division between specialists and general dentists; thirty-three (47%) wanted to increase the share of general dentists mainly by delegating simpler treatments to them: treatment with appliances such as quadhelix, headgear, activator, face mask, and removable appliances. Seventeen respondents (24%) wanted to decrease the involvement of general dentists in difficult treatments and the number of treatments started independently, without consultation.

Only one specialist orthodontist wanted to decrease delegation to auxiliaries, while 65 of them wanted to increase delegation by devolving routine tasks more often. The most common tasks to be delegated were the taking of impressions (51% of respondents answering this question), rebonding (50%), setting of bands (50%), health education and motivation (42%), bonding of brackets (15%), and changing of ligatures and arch wires (8%).

Eighty-one percent of specialist orthodontists had made some changes in their treatment practices during the preceding ten years. The most frequent changes concerned the application of new treatment techniques (71%), and of these, the adoption of an eruption guidance appliance was most common (36%). Secondly, respondents reported changes in the timing of treatment (54%), with the majority (75%) moving to an earlier starting age. Thirdly, fourteen respondents were delegating orthodontic tasks to general dentists or auxiliaries more often.

When the specialist orthodontists were asked to name those features of Finnish orthodontic care they considered to be of good quality, 55% listed the population-based system in the organization of orthodontic services, 25% the good professional skills of specialists, and 20% professional skills in the execution of early treatment.

When assessing public orthodontic care as a whole, the orthodontists complained about the wide variation in the access to orthodontic treatment and unsatisfactory routines in the treatment processes. The most frequently mentioned improvement suggestion was an increase in specialist manpower (Table 5).

Both respondent groups stressed the need for research on treatment outcome and stability of treatment results. The efficacy of treatment methods was similarly mentioned by both groups, while the need for studies on cost-effectiveness was especially emphasized by the orthodontists (Table 6).

## 4. Discussion

There was a wide variation in the extent of orthodontic services among Finnish health centres. The variation in the access and delivery of treatment also seemed to be the main concern among the orthodontists working in or cooperating with health centres. The economic depression in the 1990s had not been directly reflected in the extent of orthodontic services, and more health centres had increased than decreased their services during the last decade.

The earlier survey on public orthodontic care of children and adolescents in Finnish municipal health centres made it possible to evaluate the changes in services during the ten-year period [2]. The overall extent of services had generally increased, but the 20-fold differences among health centres still prevailed. A new appliance, the eruption guidance appliance, was introduced during the period. The eruption guidance appliance seemed to have replaced the use of removable plates and functional appliances in the early treatment group. Delegation of orthodontic tasks to auxiliaries had increased, and delegation was widely accepted by the orthodontists.

The information on orthodontic treatment delivery in health centres was collected retrospectively. The majority of the nonresponding health centres were smaller ones, which obviously had more difficulties in collecting the retrospective data. Because this evaluation concentrated on public services provided in municipal health centres, it was justified to include only the views of the orthodontists working in or cooperating with the health centres.

The methods of measuring the volume of orthodontic treatment vary in different countries, and this hampers reliable comparisons [3, 12, 13]. According to Chestnutt et al. [12], the extent of orthodontic treatment also increased in Britain between 1993 and 2003. Correspondingly, we found that the mean percentage of children and adolescents receiving orthodontic treatment had increased from 7.6% in 1992 to 11.4% in 2001 [2].

The average share of orthodontic visits of all dental visits of children and adolescents had increased slightly from 26% in 1992 [2] to 30% in this study. An explanation for the increase in this ratio is the simultaneous decrease in numbers of general dental visits [14]. Furthermore, in 1998, the National Research and Development Centre for Welfare and Health published a report recommending longer oral examination intervals in children and adolescents [15]. However, the large variation among health centres in the share of orthodontic visits (2–66%) cannot be plainly explained by this change.

Most of the changes in providing orthodontic services were related to the availability of specialist manpower; an increase in the number of orthodontists was reported more often than a lack of specialists. The possibilities to organize specialist services seem to differ between small and large health centres. When compared with the earlier study, the availability of health centres' own orthodontic expertise had slightly increased, as the percentage of health centres employing their own salaried specialist had increased from 18% to 21% [2]. All municipalities, regardless of their size, have an equal responsibility to organize treatment even in the most severe cases. This might be one reason why the percentage of health centres purchasing commissioned services had increased from 8% in 1992 to 34% in 2001.

According to the respondents in the present study, the weakened economic situation was the most frequent reason for reducing children's orthodontic services. The effects of economic restraints on the public orthodontic services have been evaluated in Denmark and in Sweden [16, 17]. According to Linder-Aronson et al. [17], the restrictions of orthodontic services cannot be defended by a decreasing need of treatment. Actually, the restriction of access to orthodontic treatment seems to lead to an increased consumption of resources [16]. In Sweden, the economic restraints seemed to decrease the number of treatments provided by general dentists and the use of appliances demanding good compliance [18].

It has been suggested that the costs of orthodontic services could be lowered by changes in the division of work and by delegation of tasks to dental auxiliaries [19]. The delegation of orthodontic tasks is an acceptable practice in all Nordic countries, but is applied most widely in Denmark and Sweden. In Sweden, the work division is also facilitated by the systematic training of orthodontic assistants [20]. In Finland, the share of health centres applying delegation of orthodontic tasks to auxiliaries had increased from 28% to 61% from 1991 to 2001 [2]. This development seemed to be largely accepted among Finnish orthodontists. They were also interested in devolving simpler orthodontic treatments to general dentists with the premise that the planning of treatment was carried out by specialists.

The orthodontists were concerned about the great variation in the delivery of orthodontic care and almost half of them suggested that national guidelines should be established to reduce this variation. The development of guidelines has also been suggested earlier as a good tool for reducing variation in the provision of health services [21, 22]. Guidelines on the screening of malocclusions and the assessment of treatment need could diminish the variation in access to and volume of treatment. Furthermore, the inefficient routines used in the planning and documentation of treatments, which were also mentioned, could be improved by national guidelines.

## 5. Conclusions

National guidelines and delegation of orthodontic tasks were suggested as principal tools for reducing the wide variation among the health centres. The retirement of orthodontists will accelerate in the near future. Thus, the orthodontists' suggestion to increase the number of specialist orthodontists is justified. Additional resources for orthodontic services should be established without further delay by organizing local and national training for auxiliary personnel working in orthodontic teams. Furthermore, development of national guidelines for the orthodontic treatment process might increase both the uniformity and effectiveness of treatment.

## Figures and Tables

**Table 1:** The volume of orthodontic services in the health centres of different size groups in 2001 measured by the number of 0–18-year-old children wearing appliances and by the share of orthodontic visits of all visits in the age group of 0–18-year-olds.

Number of inhabitants	Percentage of 0–18-year-olds wearing an orthodontic appliance	Percentage of orthodontic visits of all visits of 0–18-year-olds
	mean (SD)	mean (SD)
Below 10 000	13.6 (7.1)	30.4 (10.0)
10 000–19 999	12.8 (6.3)	30.7 (14.1)
20 000–29 999	11.9 (6.0)	31.3 (6.0)
30 000–50 000	10.6 (4.4)	32.6 (6.2)
Above 50 000	10.3 (6.4)	30.1 (8.7)

**Table:** (a) First, second, and third most frequently used orthodontic appliances at the age of 7–9 years (*N* = 205)

Appliance	First *N*	Second *N*	Third *N*
Headgear	91	59	32
Quadhelix	64	64	32
Eruption guidance appliance	34	35	41
Removable plate	6	4	8
Functional appliance	3	16	18

**Table:** (b) First, second, and third most frequently used orthodontic appliances at the age of 10–13 years (*N* = 204)

Appliance	First *N*	Second *N*	Third *N*
Headgear	91	43	27
Fixed appliance	71	69	44
Functional appliance	16	58	54
Eruption guidance appliance	13	12	20
Quadhelix	12	7	7

**Table 3:** How orthodontic expertise is obtained in the health centres of different sizes, measured as the number of inhabitants in the area.*

Size of health centre as the number of inhabitants (Number of health centres)	Own salaried specialist	Purchased consultant services	Purchased commissioned services
Below 10 000 (100)	2	91	40
10 000–19 999 (50)	6	41	15
20 000–29 999 (22)	11	11	4
30 000–50 000 (18)	10	8	4
Above 50 000 (16)	14	2	6
All (206)	43	153	69

*Some health centres obtain expert services in several ways.

**Table 4:** Changes in the volume of orthodontic services during the previous five years reported by local chief dental officers (*N* = 34).

Changes reported (*N*)	Explanations given by respondents (*N*)
Volume of orthodontic treatment increased (27)	Specialist manpower increased (17)
	Orthodontic services better organized (15)
	Commissioned services increased (4)
	More general dentists participated in orthodontic treatments (4)

Volume of orthodontic treatment decreased (7)	Weakened economic situation (5)
	Increased need for adults' dental services (4)
	Lack of specialist manpower (3)

**Table 5:** Aspects in need of improvement and suggested tools for improvement according to specialist orthodontists (percentage of respondents in parenthesis).

Aspects in need of improvement	(%)
Lack of national guidelines for orthodontic care	40
Inefficient routines in documentation, planning and follow-up of treatments	36
Insufficient work division in orthodontic care	35
Lack of orthodontic skills among general dentists	30

Suggested tools for improvement	(%)

Increased education of specialist orthodontists	39
Additional orthodontic resources needed for public health services	30
Better cooperation between central hospitals and health centres	19
Remuneration of orthodontic treatment by sickness insurance or by introducing orthodontic service vouchers for private services	16

**Table 6:** Suggestions for subjects for further research in orthodontics (percentage of respondents in parenthesis).

Suggested subject	Chief dental officers (%)	Orthodontists (%)
Long-term stability of treatment result	18	30
Effect of timing on treatment outcome	18	25
Comparison of different treatment modalities	12	10
Cost-effectiveness analysis	12	23
Need and indications for orthodontic treatment	6	5
Efficacy of eruption guidance appliances	5	7
Efficacy of different orthodontic treatment practices	5	7
